# Neural network execution using nicked DNA and microfluidics

**DOI:** 10.1371/journal.pone.0292228

**Published:** 2023-10-19

**Authors:** Arnav Solanki, Zak Griffin, Purab Ranjan Sutradhar, Karisha Pradhan, Caiden Merritt, Amlan Ganguly, Marc Riedel

**Affiliations:** 1 Department of Electrical and Computer Engineering, University of Minnesota Twin-Cities, Minneapolis, MN, United States of America; 2 Department of Computer Engineering, Rochester Institute of Technology, Rochester, NY, United States of America; Model Institute of Engineering and Technology, INDIA

## Abstract

DNA has been discussed as a potential medium for data storage. Potentially it could be denser, could consume less energy, and could be more durable than conventional storage media such as hard drives, solid-state storage, and optical media. However, performing computations on the data stored in DNA is a largely unexplored challenge. This paper proposes an integrated circuit (IC) based on microfluidics that can perform complex operations such as artificial neural network (ANN) computation on data stored in DNA. We envision such a system to be suitable for highly dense, throughput-demanding bio-compatible applications such as an intelligent Organ-on-Chip or other biomedical applications that may not be latency-critical. It computes entirely in the molecular domain without converting data to electrical form, making it a form of *in-memory* computing on DNA. The computation is achieved by topologically modifying DNA strands through the use of enzymes called nickases. A novel scheme is proposed for representing data stochastically through the concentration of the DNA molecules that are nicked at specific sites. The paper provides details of the biochemical design, as well as the design, layout, and operation of the microfluidics device. Benchmarks are reported on the performance of neural network computation.

## 1 Introduction

The biochemistry of Dioxyribonecleic Acid (DNA) has fascinated scientists for several decades. The DNA molecule is capable of storing large volumes of information and is capable of passing that information between multiple generations. The molecule is known to be stable in a huge range of environmental and temperature variations across decades, centuries and even millennia all inside molecular dimensions. Modern computing storage media is generations behind in creating a stable and durable memory substrate that is useable at scale. Recent research has demonstrated the capability of using DNA molecules to store not just genetic information but also encoded digital information [1]. Inspired by this breakthrough computer scientists have been researching way to compute with DNA molecules resulting in designs capable of performing logical operations as well as complex arithmetic such as vector-matrix operations [2–4]. Due to the ultra-compact form factor and unprecedented characteristics of the DNA molecule favoring its future use as big-data storage medium, we envision a computing platform using DNA molecules that are capable of computation in-situ without the need for domain conversion of information from DNA to electronics. It is shown in [5] how various deep learning methods in conjunction with Microfluidics can address open problems in Biotechnology. Therefore, in this paper we present a novel method for implementing mathematical operations in general, and artificial neural networks (ANNs) in particular, with molecular reactions on DNA in a microfluidic device. While biochemical reaction representing computations using DNA molecules are several orders of magnitude slower than electronic gates, their data density is 3 orders of magnitude higher and 8 orders of magnitude lower in energy consumption than solid state memory [6]. Therefore, applications such as archival information processing, search and recognition tasks that are not latency critical but need high physical density and massive parallel throughput as well as bio-compatibility are suitable applications for this technology. Such a system can be inserted into living tissue and organic matter for augmenting, memory, intelligence and processing capacity in a future world (for example, Neuralink). When coupled with biotechnological innovations, DNA based neural network computing can provide intelligence to an Organ-on-Chip [7].

In what follows, we discuss the impetus to store data and perform computation with DNA. Then we outline the microfluidic technology that we will use for these tasks.

### 1.1 Background

The fields of *molecular computing* and *molecular storage* are based on the quixotic idea of creating molecular systems that perform computation or store data directly in molecular form. Everything consists of molecules, of course, so the terms generally mean computing and storage in *aqueous* environments, based on chemical or biochemical mechanisms. This is in contrast to conventional computers, in which computing is effected *electrically* and data is either stored *electrically* (in terms of voltage) in solid-state storage devices, *magnetically* in hard drives, or *optically* on CDs and DVDs. Given the maturity of these conventional forms of computing and storage, why consider chemical or biochemical means?

The motivation comes from distinct angles:

1. Molecules are very, very ***small***, even compared to the remarkable densities in our modern electronic systems. For instance, DNA has the potential to store approximately 1,000 times more data per unit volume compared to solid-state drives. Small size also means that molecular computing can be *localized*, allowing it to be performed in confined spaces, such as inside cells or on tiny sensors.
2. In principle, molecular computing could offer unprecedented ***parallelism***, with billions of operations occurring simultaneously.
3. Molecular computing has the potential to consume much less ***energy*** than our silicon systems, which always require a bulky battery or wired power source.
4. The use of naturally occurring molecules with enzymes results in a more ***sustainable*** computer design, without the use of toxic and unethically sourced raw materials.
5. Finally, molecular computing could be deployed ***in situ*** in our bodies or our environment. The goal here is to perform sensing, computing, and actuating at a molecular level, with no interfacing at all with external electronics. The inherent biocompatibility of molecular computing components offers the possibility of seamless integration into biological systems.

### 1.2 DNA storage

The leading contender for a molecular storage medium is DNA. Ever since Watson and Crick first described the molecular structure of DNA, its information-bearing potential has been apparent to computer scientists. Each nucleotide in the sequence is drawn from the four-valued alphabet {*A*, *T*, *C*, *G*}, and a DNA molecule with *n* nucleotides stores 4^*n*^ bits of data. In fact, this information storage underpins life as we know it: all the instructions on how to build and operate a life form are stored in its DNA, honed over eons of evolutionary time.

In a highly influential 2012 Science paper, the renowned Harvard genomicist George Church made the case that we will eventually turn to DNA for information storage, based on the ultimate physical limits of materials [8]. He delineated the theoretical storage **capacity** of DNA: 200 petabytes per gram; the read-write **speed**: less than 100 microseconds per bit; and, most importantly, the **energy** required: as little as 10^−19^ joules per bit. This energy requirement is orders of magnitude below the femtojoules/bit (10^−15^ J/bit) barrier touted for other emerging technologies. Furthermore, DNA remains stable for decades, perhaps even millennia, as evidenced by DNA extracted from the carcasses of woolly mammoths. In principle, DNA could outperform all other types of media that have been studied or proposed.

However, no DNA storage system has yet been built that comes close to beating existing media (magnetic, optical, or solid-state storage). The practical challenges are formidable. Fortunately, DNA technology is not exotic. Driven by the biotech and pharma industries, the technology for both sequencing (*reading*) and synthesizing (*writing*) DNA has followed a trajectory similar to Moore’s law over the past 20 years. DNA strands can be synthesized for less than $0.50 per base pair, and the sequencing of a whole genome can be completed within 6 hours. Undoubtedly inspired by Church’s first-principles thinking, and motivated by the trajectory of sequencing and synthesis technology, there has been a groundswell of interest in DNA storage. The leading approach involves DNA synthesis based on phosphoramidite chemistry [9]. However, numerous other creative ideas and novel technologies, ranging from nanopores [10] to DNA origami [11], are also being explored.

### 1.3 DNA computing

Beginning with the seminal work of Adelman a quarter-century ago [12], DNA computing has promised the benefits of massive parallelism in operations. These operations are typically performed on the *concentration* of DNA strands in a solution. For instance, using DNA strand displacement cascades, single strands displace segments of double strands, thereby releasing single strands that can subsequently participate in further operations [13–15]. Both the inputs and outputs correspond to the concentration values of specific strands.

It is fair to say that in the three decades since Adelman first proposed the idea, the practical impact of research on this topic has been modest. However, a practical DNA storage system, particularly one that is inherently programmable, has the potential to change this scenario. Such storage opens up the possibility of ‘in-memory’ computing, which involves performing computation directly on the data stored in DNA [2, 16, 17]. This computation is not performed on the sequence of nucleotides, but rather by making topological modifications to the DNA strands: introducing breaks in the phosphodiester backbone known as ‘nicks’ and gaps in the backbone referred to as ‘toeholds’. Enzymatic processes, such as CRISPR/Cas9, can be used for the nicking process [18, 19].

**Fig 1:**
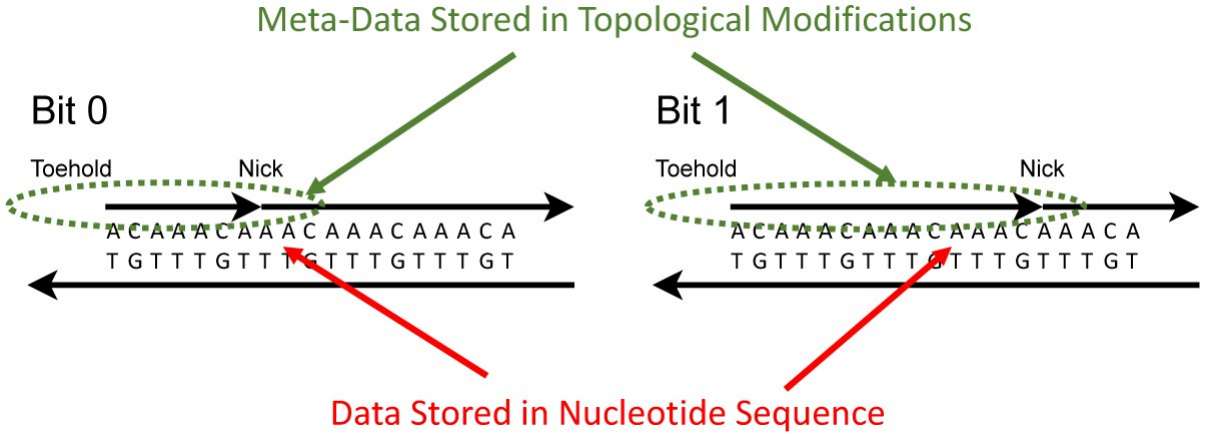
Data is stored in multiple dimensions. The sequence of nucleotides stores data in the form of the *A*’s, *C*’s, *T*’s, and *G*, with 2 bits per letter. Superimposed on this, we store data via topological modifications to the DNA, in the form of nicks and exposed toeholds. This data is **rewritable**, with techniques developed for DNA computation.

It is important to note that the data used for this form of DNA computing is encoded in a different dimension compared to the sequence data of the DNA. The **underlying data**—which could amount to terabytes—is stored as the sequence of *A*’s, *C*’s, *T*’s, and *G*’s in synthesized strands. Superimposed on this data, we store **metadata** using topological modifications. This concept is illustrated in Fig 1. The metadata is rewritable and thus aligns with the paradigm of in-memory computing [20]. The computation follows a Single Instruction, Multiple Data (SIMD) form. SIMD is a computer engineering acronym for Single Instruction, Multiple Data [21], representing a form of computation in which multiple processing elements perform the same operation on multiple data points simultaneously. This is in contrast to the more general class of parallel computation called MIMD (Multiple Instructions, Multiple Data). Much of the modern progress in electronic computing power has come from scaling up SIMD computation with platforms like graphical processing units (GPUs). SIMD provides a means to transform stored data—potentially large amounts of it—with a single parallel instruction.

### 1.4 Stochastic logic

The form of molecular computing that we present in this paper is predicated on a novel encoding of data. A link is made between the representation of random variables with a paradigm called *stochastic logic* on the one hand, and the representation of variables in molecular systems as the concentration of molecular species, on the other.

**Fig 2:**
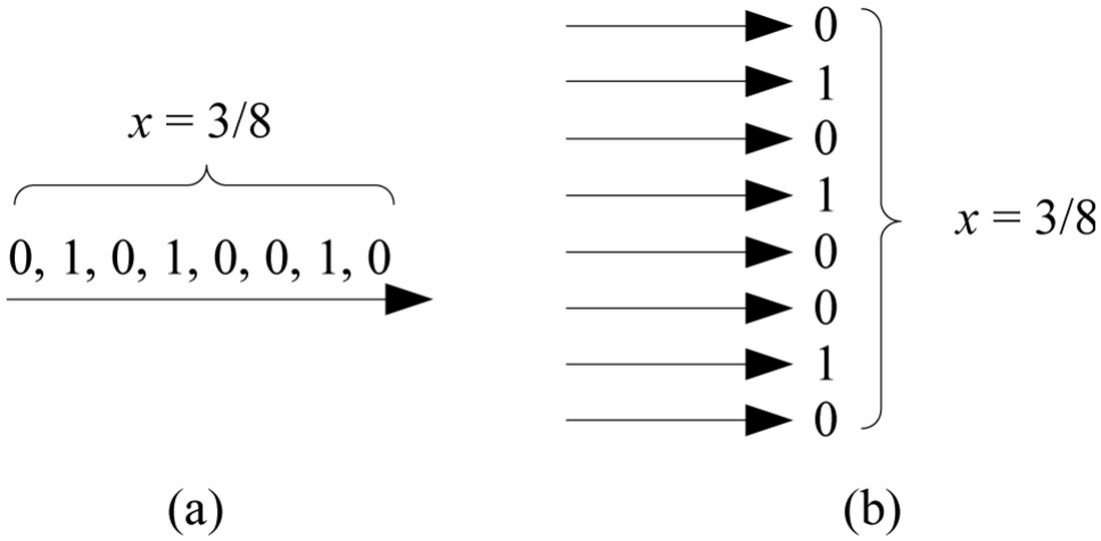
Stochastic representation: A random bitstream. A value *x* ∈ [0, 1], in this case 3/8, is represented as a bitstream. The probability that a randomly sampled bit in the stream is one is *x* = 3/8; the probability that it is zero is 1 − *x* = 5/8.

Stochastic logic is an active topic of research in digital design, with applications to emerging technologies [22–24]. Computation is performed with familiar digital constructs, such as AND, OR, and NOT gates. However, instead of having specific Boolean values of 0 and 1, the inputs are random bitstreams. A number *x* (0 ≤ *x* ≤ 1) corresponds to a sequence of random bits. Each bit has *probability* *x* of being one and probability 1 − *x* of being zero, as illustrated in Fig 2. Computation is recast in terms of the probabilities observed in these streams. Research in stochastic logic has demonstrated that many mathematical functions of interest can be computed with simple circuits built with logic gates [23, 25].

Consider basic logic gates. Given a stochastic input *x*, a NOT gate implements the function

$$\begin{array}{c} \text{NOT}(x)=1-x. \end{array}$$ (1)

This means that while an individual input of 1 results in an output of 0 for the NOT gate (and vice versa), statistically, for a random bitstream that encodes the stochastic value *x*, the NOT gate output is a new bitstream that encodes 1 − *x*. The output of an AND gate is 1 only if all the inputs are simultaneously 1. The probability of the output being 1 is thus the probability of all the inputs being 1. Therefore, an AND gate implements the stochastic function:

$$\begin{array}{c} \text{AND}(x,y)=xy, \end{array}$$ (2)

that is to say, multiplication. Probabilities, of course, are values between 0 and 1, inclusive. If we express them as rational numbers, given some positive integer *n* as the denominator, we have fractions

$$\begin{array}{c} x=\frac{a}{n},\;y=\frac{b}{n} \end{array}$$

where 0 ≤ *a* ≤ *n* and 0 ≤ *b* ≤ *n*. So an AND gate computes *a fraction of a fraction.*

We can implement other logic functions. The output of an OR gate is 0 only if all the inputs are 0. Therefore, an OR gate implements the stochastic function:

$$\begin{array}{c} \text{OR}(x,y)=1-(1-x)(1-y)=x+y-xy. \end{array}$$ (3)

The output of an XOR gate is 1 only if the two inputs *x*, *y* are different. Therefore, an XOR gate implements the stochastic function:

$$\begin{array}{c} \text{XOR}(x,y)=(1-x)y+x(1-y)=x+y-2xy. \end{array}$$ (4)

The NAND, NOR, and XNOR gates can be derived by composing the AND, OR, and XOR gates each with a NOT gate, respectively. Please refer to Table 1 for a full list of the algebraic expressions of these gates. It is well known that any Boolean function can be expressed in terms of AND and NOT operations (or entirely in terms of NAND operations). Accordingly, any function can be expressed as a nested sequence of multiplications and 1 − *x* type operations.

**Table 1:** Stochastic function implemented by basic logic gates.

gate	inputs	function
NOT	*x*	1 − *x*
AND	*x*, *y*	*xy*
OR	*x*, *y*	*x* + *y* − *xy*
NAND	*x*, *y*	1 − *xy*
NOR	*x*, *y*	1 − *x* − *y* + *xy*
XOR	*x*, *y*	*x* + *y* − 2*xy*
XNOR	*x*, *y*	1 − *x* − *y* + 2*xy*

There is a large body of literature on the topic of stochastic logic. We point to some prior work in this field. In [26] we proved that any multivariate polynomial function with its domain and codomain in the unit interval [0, 1] can be implemented using stochastic logic. In [23], we provided an efficient and general synthesis procedure for stochastic logic, the first in the field. In [27], we provided a method for transforming probabilities values with digital logic. Finally, in [28, 29] we demonstrated how stochastic computation can be performed deterministically.

### 1.5 DNA strand displacement

DNA is generally present in double-stranded form (**dsDNA**), in double-helix, with A’s pairing with T’s, and C’s with G’s. Without qualification, when we refer to “DNA” we mean double-stranded. However, for the operation we describe here, DNA in single-stranded form (**ssDNA**) plays a role.

The molecular operation that we exploit in our system is called DNA strand displacement [13, 30]. It has been widely studied and deployed. Indeed, prior work has shown that such a system can emulate *any* abstract set of chemical reactions. The reader is referred to Soloveichik et al. and Zhang et al. for further details [14, 31]. Here we illustrate a simple, generic example. In Section 4, we discuss how to map our models to such DNA strand-displacement systems.

We begin by first defining a few basic concepts. DNA strands are linear sequences of four different nucleotides {*A*, *T*, *C*, *G*}. A nucleotide can bind to another following *Watson-Crick* base-pairing: A binds to T, C binds to G. A pair of single DNA strands will bind to each other, a process called *hybridization*, if their sequences are complementary according to the base-pairing rule, that is to say, wherever there is an *A* in one, there is a *T* in the other, and vice versa; and whenever there is a *C* in one, there is a *G* in the other and vice-versa. The binding strength depends on the length of the complementary regions. Longer regions will bind strongly, smaller ones weakly. Reaction rates match binding strength: hybridization completes quickly if the complementary regions are long and slowly if they are short. If the complementary regions are very short, hybridization might not occur at all. (We acknowledge that, in this brief discussion, we are omitting many relevant details such as temperature, concentration, and the distribution of nucleotide types, i.e., the fraction of paired bases that are A-T versus C-G. All of these parameters must be accounted for in realistic simulation runs.)

**Fig 3:**
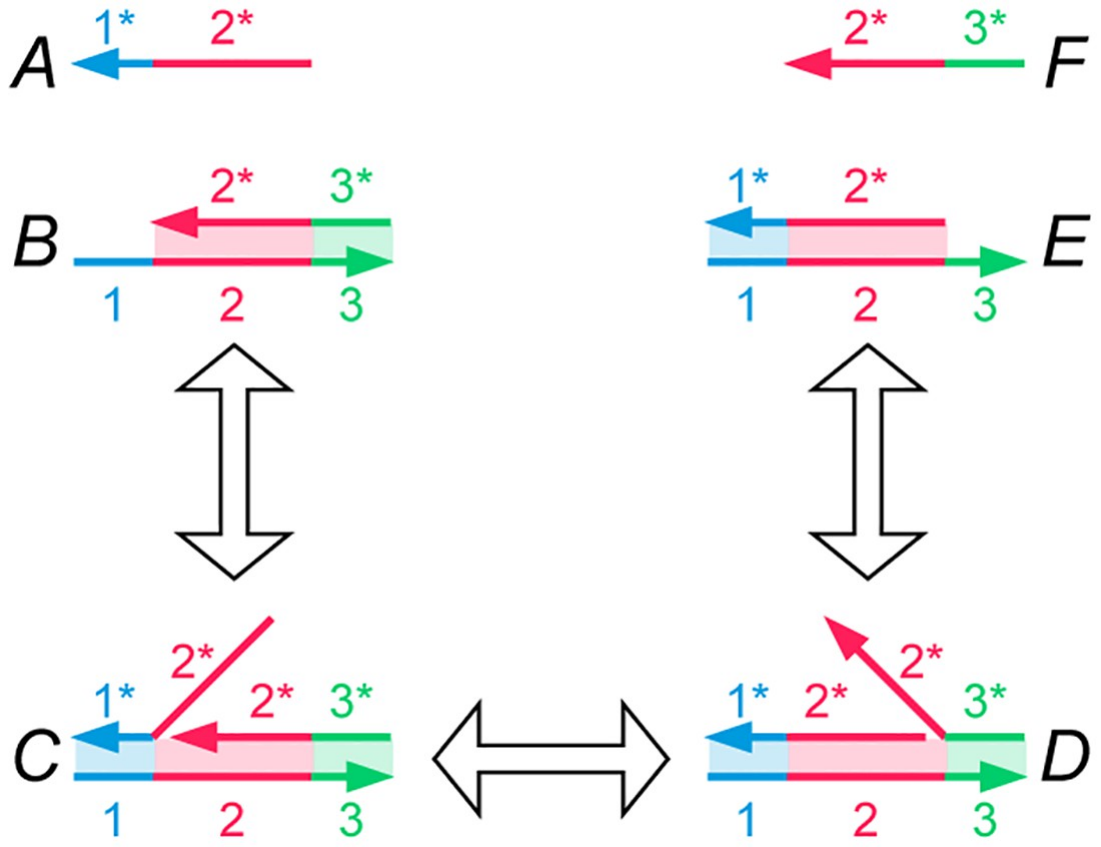
A set of DNA strand displacement reactions. Each DNA single strand is drawn as a continuous arrow, consisting of different colored domains numbered 1 through 3. DNA domains that are complementary to each other due to A–T, C–G binding are paired as 1 and 1*. The first reaction shows reactants A and B hybridizing together via the toehold at domain 1* on molecule *B*. The second reaction depicts branch migration of the overhanging flap of DNA in molecule *C*, thereby resulting in the nick migrating from after domain 1 to 2. The third reaction shows how an overhanging strand of DNA can be peeled off of molecule *D*, thereby exposing a toehold at domain 3* on molecule *E* and releasing a freely floating strand *F*. All reactions are reversible. The only domains that are toeholds are 1* and 3*.

Fig 3 illustrates strand displacement with a set of reversible reactions. The entire reaction occurs as reactant molecules *A* and *B* form products *E* and *F*, with each intermediate stage operating on molecules *C* and *D*. In the figure, *A* and *F* are single strands of DNA, while *B*, *C*, *D*, and *E* are double-stranded complexes. Each single-strand DNA molecule is divided, conceptually, into subsequences that we call **domains**, denoted as 1, 2, and 3 in the figure. The complementary sequences for these domains are 1*, 2* and 3*. (We will use this notation for complementarity throughout.) All distinct domains are assumed to be *orthogonal* to each other, meaning that these domains do not hybridize.

**Toeholds** are a specific kind of domain in a double-stranded DNA complex where a single strand is exposed. For instance, the molecule *B* contains a toehold domain at 1* in Fig 3. Toeholds are usually 6 to 10 nucleotides long, while the lengths of regular domains are typically 20 nucleotides. The exposed strand of a toehold domain can bind to the complementary domain from a longer ssDNA, and thus toeholds can trigger the binding and displacement of DNA strands. The small length of the toehold makes this hybridization reversible.

In the first reaction in Fig 3, the open toehold 1* in molecule *B* binds with domain 1 from strand *A*. This forms the molecule *C* where the duplicate 2 domain section from molecule *A* forms an overhanging flap. This reaction shows how a toehold triggers the binding of DNA strands. In molecule *C*, the overhanging flap can stick onto the complementary domain 2*, thus displacing the previously bound strand. This type of branch migration is shown in the second reaction, where the displacement of one flap to the other forms the molecule *D*. This reaction is reversible, and the molecules *C* and *D* exist in a dynamic equilibrium. The process of branch migration of the flap is essentially a random walk: at any time when part of the strand from molecule *A* hybridizes with strand *B*, more of *A* might bind and displace a part of *F*, or more of *F* might bind and displace a part of *A*. Therefore, this reaction is reversible. The third reaction is the exact opposite of reaction 1—the new flap in molecule *D* can peel off from the complex and thus create the single-strand molecule *F* and leave a new double-stranded complex *E*. Molecule *E* is similar to molecule *B*, but the toehold has migrated from 1* to 3*. The reaction rate of this reaction depends on the length of the toehold 3*. If we reduce the length of the toehold, the rate of reaction 3 becomes so small that the reaction can be treated as a forward-only reaction. This bias in the direction of the reaction means that we can model the entire set of reactions as a single DNA strand displacement event, where reactants *A* and *B* react to produce *E* and *F*. Note that the strand *F* can now participate in further toehold-mediated reactions, allowing for cascading of such these DNA strand displacement systems.

By using orthogonal domains to construct DNA molecules, high specificity can be maintained for these toeholds reactions. That is, DNA molecules will not cross react with other molecules unless they share the same toehold domains. This prevents the formation of unexpected reaction products and reduces the reaction error rate. Furthermore, these molecules can be stored in aqueous conditions for at least a year [32]. Thus toehold mediated strand displacement provides a means of designing stable and specific molecular reactions.

### 1.6 Chemical model

Recent research has demonstrated how data can be encoded via *nicks* on DNA using gene-editing enzymes like CRISPR-Cas9 and PfAgo [33]. *Probabilistic switching* of concentration values has also been demonstrated within the DNA computing community [34]. In our previous work, we showcased the adaptation of a concept from computer engineering, called *stochastic logic*, for DNA computing [35]. In this paper, we unify these distinct threads by demonstrating how to perform stochastic computation on *fractionally-encoded* data stored on nicked DNA.

The conventional approach to DNA data storage involves using a single species of strand to represent a value. This can either be done by encoding binary values, where the presence of the specific strand represents a 1 and its absence a 0 [30], or by employing a non-integer value encoding based on the concentration, referred to as a *direct representation* [36]. Recent research has explored the use of a *fractional representation* [2, 35, 37]. The central idea is to use the concentrations of two species of strand, *X*_0_ and *X*_1_, to represent a value *x* with the relationship:

$$\begin{array}{c} x=\frac{X_{1}}{X_{0}+X_{1}} \end{array}$$

where *x* ∈ [0, 1]. This encoding concept is connected to *stochastic logic*, where computation is carried out on randomized bit streams and values are represented by the fraction of 1’s versus 0’s in the stream [22, 23, 38].

In this work, values are stored based on nicking sites on double DNA strands. For a given site, some strands will be nicked, while others will not. The overall concentration of the double strand is denoted as *C*_0_, and the concentration of strands nicked at the site is denoted as *C*_1_. The ratio of the concentration of nicked strands to the overall concentration can be expressed as:

$$\begin{array}{c} x=\frac{C_{1}}{C_{0}} \end{array}$$

This ratio represents the relative concentration of the nicked strand at that specific site and is used to represent a variable *x* ∈ [0, 1].

This ratio can be established using two potential methods. One approach involves nicking a site using a gene-editing guide that is not fully complementary to the nicking site. The level of complementarity would govern the rate of nicking and subsequently set the relative concentration of nicked strands. A simpler method involves splitting the initial strand-containing solution into two samples, nicking all the strands in one sample, and then mixing the two samples to achieve the desired ratio *x*.

### 1.7 Microfluidics and Lab-on-Chip

Microfluidics is a rapidly developing discipline where small volumes of fluids are manipulated and transferred over channels whose dimensions range from one to hundreds of microns [39, 40]. Typically, such channels leverage principles of fluid dynamics enabling the modeling and design of systems where small volumes of fluids are moved to achieve a variety of purposes such as information and energy transfer. Due to their small form factors and need for very small amounts of fluids, this discipline is finding application in a variety of application domains such as cell sorting, DNA analysis, chemical synthesis and medical applications.

Utilizing the advances in microfluidics a practical device concept was envisioned as a Lab-on-Chip (LoC) [41]. A LoC is a device consisting of a network of microfluidic channels and microcells capable of transferring fluids to perform several functions such as chemical analysis, reactions, and sorting. Typical applications were in the area of medical sciences where small amounts of samples were needed to perform tests and diagnoses [41]. While the dominant application area of LoCs remains efficient medical diagnoses, advances in manufacturing capability using Integrated Circuit (IC) fabrication methodologies or 3D printing their applicability is expanding into sensing and processing more widely. In this paper, we envision a LoC device enabled by microfluidics to perform neural network computations using DNA molecules as the medium. Here we discuss the specific processes that will be required to perform the Neural Network computations using DNA molecules in a LoC.

The microfluidic mechanisms required include droplet formation, droplet movement, valving, mixing, and separation. These techniques have been extensively studied and developed in the literature. Here, we provide selected citations for the specific mechanisms essential to our proposed system.

Droplet formation can be achieved by adopting the single emulsion technique based on hydrodynamic principles, as discussed in [42]. This technique involves multiplexing liquid/emulsion flow and air or oil flow from different capillary tubes to create air/oil gaps between the liquid droplets [43]. The activation and deactivation of air/oil flow are controlled by pneumatic valves that regulate the nozzle outlets for the liquid or air flows. Encapsulating the droplets in oil prevents droplet evaporation. In our proposed system, these droplets will contain DNA molecules or nickases solutions. After droplet generation, the droplets are transported through microchannels to the microchamber array. This movement is achieved by reopening the valves to allow air flow, inducing the droplets to move due to pneumatic effects. We utilize the regular array arrangement of the destination microchambers to generate the droplets at regular intervals and propel them along the microchannels, ensuring they reach their respective microchamber entry valves simultaneously. This synchronization enables operations like a single MAC operation (mixing DNA solution with nickase solution in multiple microchambers in a single row) to occur collectively.

It’s noteworthy that within these dimensions and speeds, typically ranging from 0.1 to 0.01 mm/s, the Reynolds Number for aqueous solutions remains below 2000, resulting in laminar flow rather than turbulent flow [44]. Similarly, in the microfluidic regime, the Peclet Number is below 1 due to fluid velocities of around 0.01 mm/s, implying that the liquid flow is dominated by diffusion rather than turbulence. This emphasizes that mixing between different solutions for reactions is predominantly reliant on diffusive processes rather than convective ones. Thus, to ensure an adequate reaction time, we mix DNA and nickase solutions within microchambers to allow sufficient time for reaction completion [4]. This mixing occurs within microchambers, not while droplets are in motion through microchannels. Diffusion-dominated mixing within the microchambers is allowed to proceed to completion before the resultant droplets are moved to the next microchamber using valve and air flow control, facilitating the continuation of subsequent computations. Separation or filtration is achieved by immobilizing DNA molecules using magnetic beads within a microchamber while the liquids are in motion.

Recent advancements in fabrication techniques, as noted in [45], suggest that all these components can be feasibly fabricated using lithography techniques, which are the gold standard for microfluidic LoC fabrication.

### 1.8 Organization

The rest of this paper is organized as follows. Section 2 describes how we implement our core operation, namely multiplication. We do so by computing a *fraction* of a *fraction* of concentration values. Section 3 presents the architecture of the microfluidic system that we use to implement computation on data stored in DNA. Section 4 discusses the implementation of an artificial neural network (ANN) using our microfluidic neural engine. Section 5 simulations results of the ANN computation. Finally, Section 6 presents conclusions and discusses future work.

## 2 Multiplication

The core component of our design is the multiplication operation, computed as a fraction of a fraction of a concentration value of nicked DNA.

### 2.1 Encoding scheme

Nicking enzymes such as CRISPR-Cas9 can be used to effectively “nick” dsDNA at a particular site [18, 19, 46]. Since DNA is double-stranded, with strong base pairing between the A’s and T’s and the C’s and G’s, the molecule does not fall apart. Indeed, the nicking can be performed at multiple sites, and this process can be conducted independently. Single sided nicks on a double helix DNA backbone are stable and do not contribute to degradation of the DNA molecule [47].

Suppose a molecule of DNA molecule with a particular nicking site labeled *A* is in a solution. We separate the solution into two parts with a volume ratio *a* to 1 − *a* for some fraction *a*. Now site *A* is nicked on all DNA molecules in the first solution, while the second solution is left untouched. These two solutions are mixed back to obtain a single solution. Some molecules in this solution are nicked, while others are not. The relative concentration of DNA molecules with a nick at site *A* is *a*, while that of the molecules that are not nicked is 1 − *a*. Thus, any arbitrary fraction *a* can be encoded in a solution of DNA molecules with a nicking site. In our framework, the stochastic value encoded at a particular site in DNA is the relative concentration (between 0 and 1) of DNA molecules with a nick at that site.

An important consideration in our approach is that the nicking enzymes used must be highly site specific. Consider CRISPR Cas9, which can be guided to nick specific sites on a DNA strands by using a guide Ribonucleic acid (gRNA) molecule [48]. CRISPR Cas9 has a tolerance for single base pair mismatches when guided to a particular DNA site using RNA [49]. However, all our DNA domains are designed to be orthogonal to each other—they differ by several nucleotides. This ensures high site specificity when using CRISPR Cas9 for nicking. Thus by careful design of the DNA sequence used to store data, the nicking efficiency of CRISPR Cas9 can be optimized for our purposes. Furthermore, CRISPR Cas9 nickases have exhibited low off-target effects in *in vivo* studies [48]. As our approach is based on *in vitro* DNA editing, cellular processing (such as DNA repair mechanisms or DNA packaging) will not interfere with this efficiency.

### 2.2 Multiplying two values

**Fig 4:**
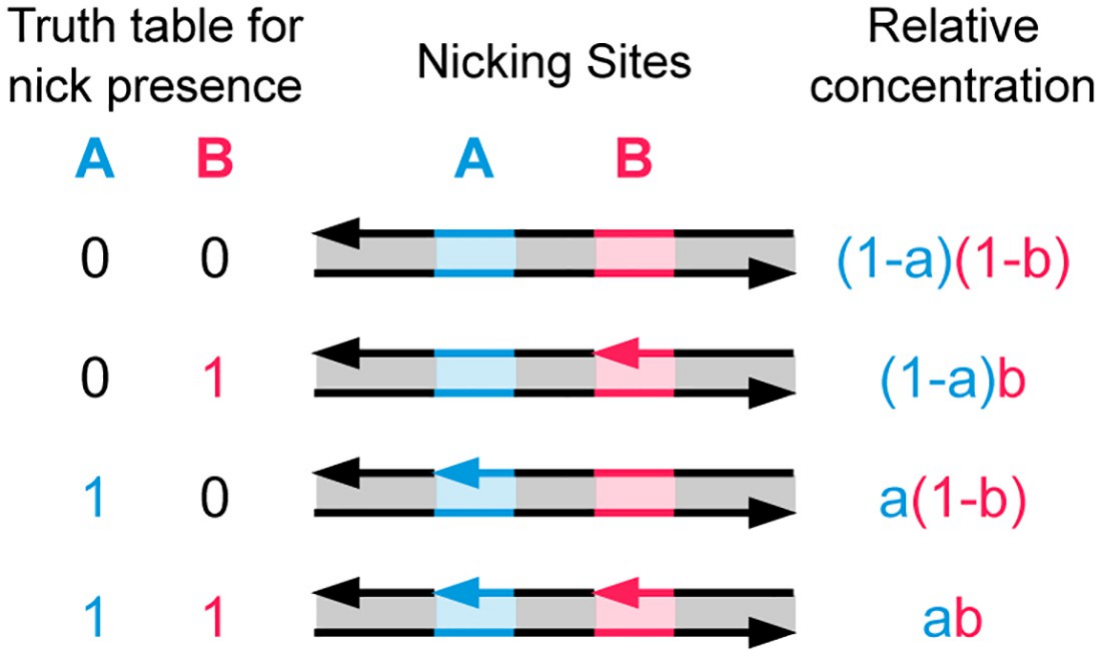
Multiplying two values, *a* and *b*, through nicking DNA. We start with a solution containing the DNA molecule shown on the top row. Fractional amount *a* of these molecules are nicked at site *A*, and *b* amount of all DNA molecules are nicked at site *B*. This results in a solution of 4 different possible DNA molecule types (as shown on each row). Assuming independent nicking on both sites, the concentration of each of these molecules is shown on the right. The molecule with nicks on both sites *A* and *B* has a concentration of *a* × *b*, that is, the product of the two fractions.

Consider a DNA molecule with two unique nicking sites, *A* and *B*. First, a stochastic value *a* is encoded at site *A*, as was discussed in Section 2.1. Now the single solution is again split into two parts, of volume ratio *b* to 1 − *b*. All molecules are nicked at site *B* in the first solution, while the second solution is again left untouched. Mixing these two solutions yields a solution containing DNA molecules that are either nicked at site *B* or not. Thus, site *B* now encodes the stochastic value *b*. Now both sites *A* and *B* are being used to independently store stochastic values *a* and *b*. Since either site could be nicked or not nicked, there are 4 different possible molecules, as shown in Fig 4. Most significantly, the molecule containing two nicks, both at site *A* and *B*, has a relative concentration of *a* × *b*. That is the product of the two fractional values—a fraction of a fraction. The concentrations of all other molecules are also listed in Fig 4. Note that these values only hold if both sites are nicked independently.

Thus, our encoding approach not only allows us not only to store data but also to compute on it. This is ideal for computing a scalar multiplication in a neural network—input data is initialized at site *A* in a given solution, and then the scalar weight it is to be multiplied with is stored at site *B*. In this approach, it is necessary for sites *A* and *B* to be neighboring each other (i.e., no other nicking sites lie between them) to allow for readout.

### 2.3 Reading out

Having covered storing two stochastic values in a single solution, we now discuss multiplying these values.

**Fig 5:**
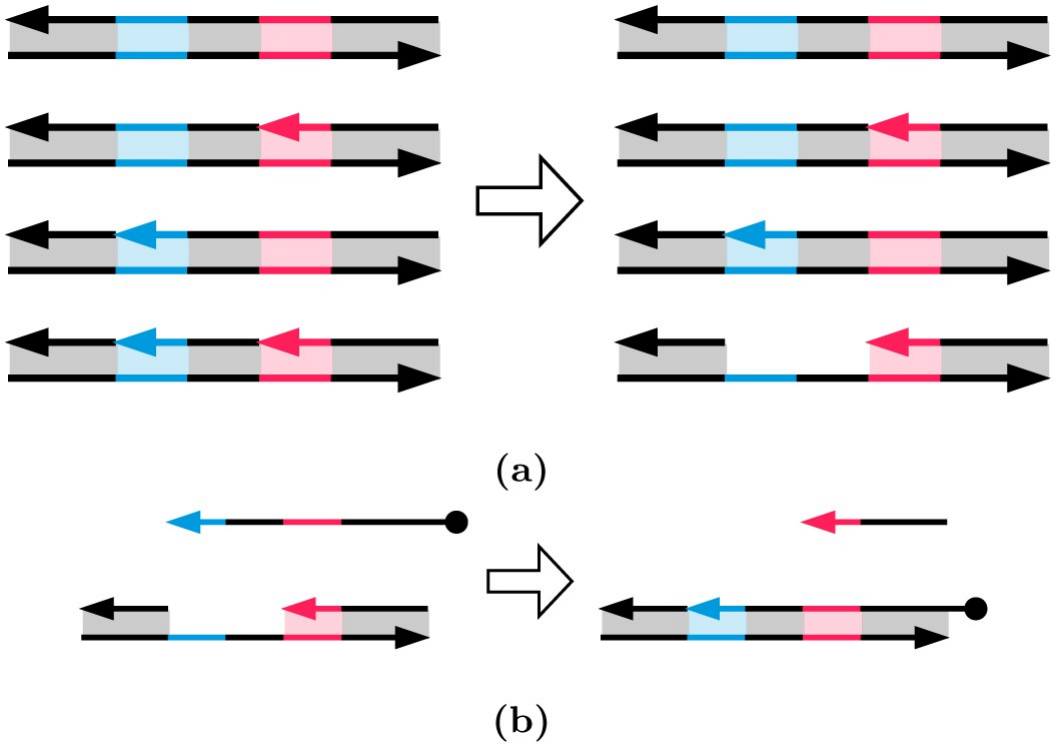
Reading out the multiplication results. (a) The DNA solution storing stochastic values *a* and *b* on sites *A* and *B* is gently heated. This creates a toehold only on the molecules with nicks on both sites, i.e., the *a* × *b* molecules. (b) A probe strand (the first reactant) can then bind with the newly exposed toehold and displace ssDNA (the first product). The concentration of this ssDNA stores the product *a* × *b*.

Assume a solution storing two stochastic values *a* and *b*, as detailed in Section 3.2. This solution is gently heated to initiate denaturing of DNA. That is, the DNA starts to break apart into two strands. By restricting the temperature, only short regions with low G-C content will fully denature, while longer strands remain bound. For our starting molecule, the short region between the nicking sites *A* and *B* will fully break apart into a single-stranded region. That is, a toehold will be formed between these two sites [50]. This toehold will only be formed on DNA molecules with nicks on both sites, so only *a* × *b* amount of molecules will have a toehold. Now a probe strand is supplied that will bind to the newly exposed toehold. This probe strand is used to displace the DNA strand adjacent to the toehold. The amount of single-stranded DNA (ssDNA) that is displaced through this process is again *a* × *b* the amount of the starting dsDNA. Thus, the product of two stochastic variables can be read out *in vitro*. This procedure is shown in Fig 5. In Section 4, we discuss how these single strands can then participate in further strand-displacement operations.

**Fig 6:**
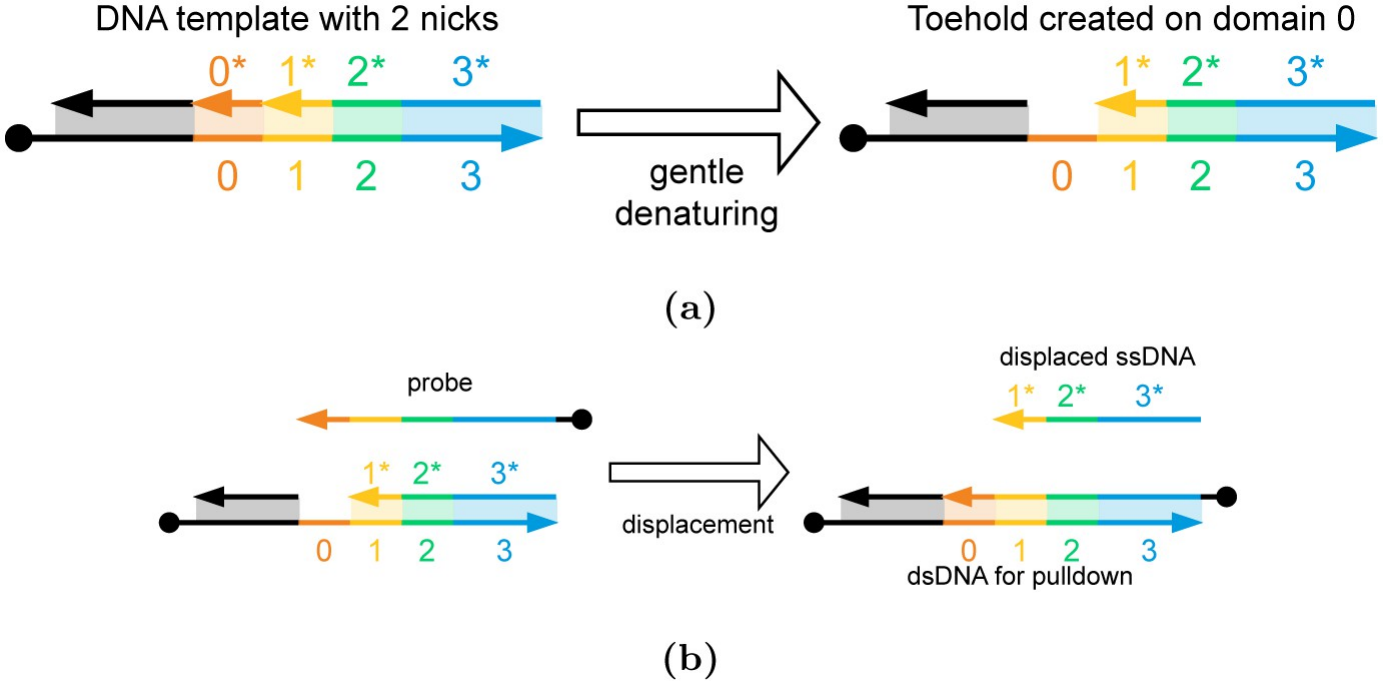
Extracting ssDNA from dsDNA molecules using probe strands. (a) The DNA template molecule with two nicks at sites *A* and *B*. After applying gentle heat, the ssDNA between the two nicks is selectively denatured to create a toehold at domain 0. (b) A probe strand is used to displace the ssDNA spanning domains 1 to 3 from the DNA molecule. The ssDNA is separated from all the other DNA molecules (i.e., the DNA and any excess probe strands) as the other molecules can all be pulled out.

It is important to cleanly separate the dsDNA molecules from the ssDNA extracted above. To achieve this, the dsDNA molecules and probe strands can have magnetic beads attached to them [51–54]. When a magnetic field is applied to the solution, the dsDNA molecules and any excess probe strands can be pulled down from the solution, allowing the displaced ssDNA to be separated. These magnetic beads are shown in Fig 6.

## 3 DNA-based neural engine

**Fig 7:**
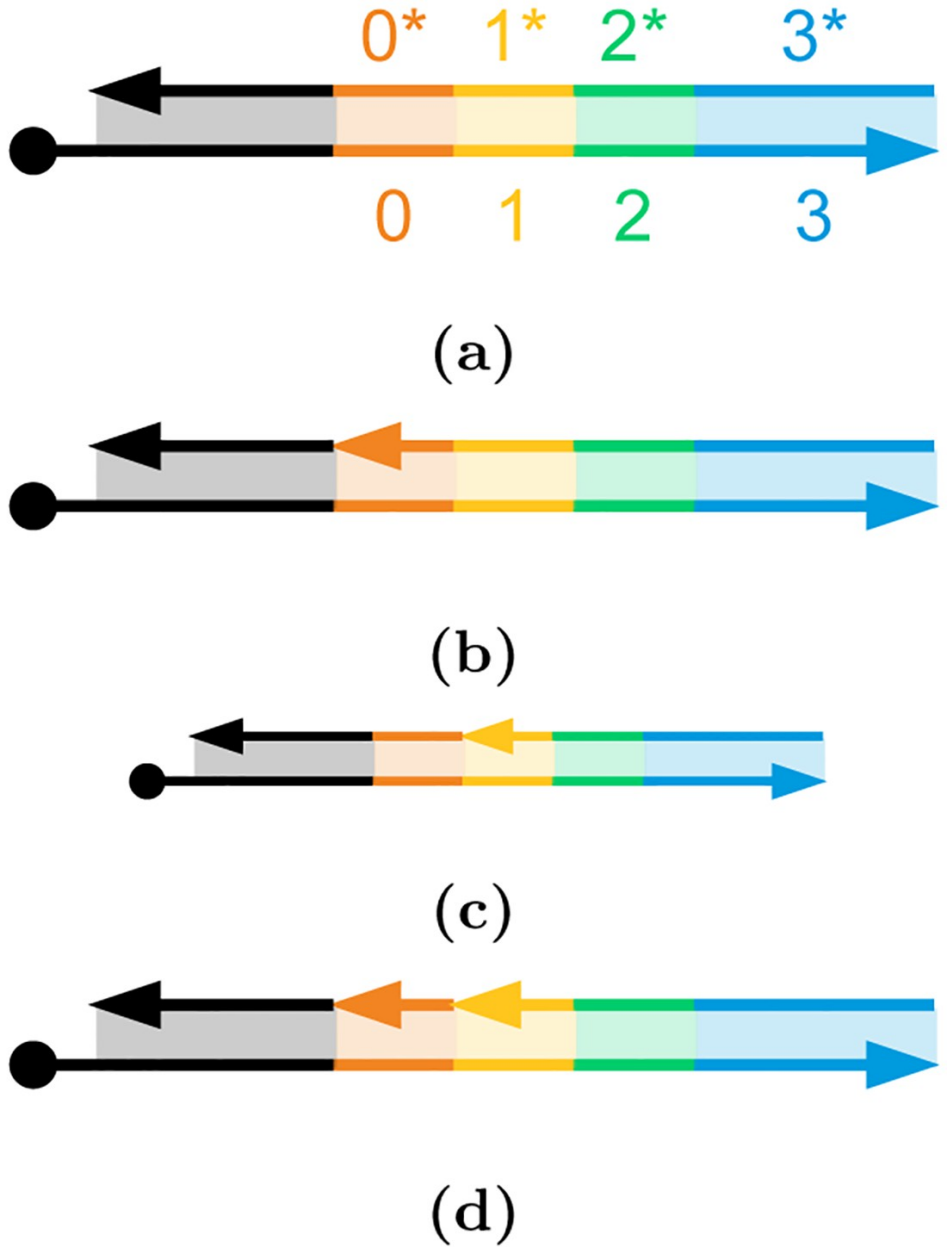
Storing data on DNA molecules using nicks. (a) The DNA template molecule consists of domains 0 to 4 (in color), with an additional unnamed domain (black) preceding them and a magnetic bead attached (on the left). 0*-4* denote the complementary top strand sequence for these domains. (b) The DNA molecule with a nick at nicking site *A* between the black domain and 0*. (c) The DNA molecule with a nick at nicking site *B* between the 0* and 1*. (d) The DNA with nicks on both nicking sites. Only this DNA molecule with two nicks represents data value 1; the other three configurations (a)-(c) correspond 0.

ANN computational workload consists primarily of matrix operations and activation functions. Among the matrix operations, matrix-matrix multiplication (GEMM) and matrix-vector multiplication (GEMV) make up almost the entirety of the workload which can be performed via repeated multiplications and accumulations (MAC). In the proposed DNA Neural Engine the process of performing a multiplication will take advantage of the stochastic representation of the operands. The input to a single neuron can be stochastically represented by the proportion of DNA strands nicked at a consistent site, compared to the total number of DNA strands in a solution (*i.e.,* the concentration of specifically nicked DNA strands). In this paper, molecules with 2 nicks as shown in Fig 7 represent value 1, while all other molecule types correspond to 0. The relative concentration of doubly-nicked DNA molecules is the stochastic value stored in the solution.

The neuron weights, on the other hand, are represented by the concentration of enzymes in a droplet intended to create a second nick on the already-nicked DNA molecules. To perform the stochastic multiplication for each neuron’s input-weight pair, the droplet with a concentration of enzymes, representing the weight value, is mixed with the droplet of the nicked DNA strands to create a second nick in the DNA strands. The second nicking site is required to be within around 18 base pairs of the first nick to allow a small fragment between the two nicked sites to be detached upon the introduction of probe strands. The product of the input and weight for this particular neuron is represented by the relative concentration of double-nicked strands compared to the total concentrations of DNA strands.

It may be noted that at the beginning of the processing, the inputs to the neural engine may also be set by this multiplication process where a solution of un-nicked DNA strands are nicked in a single site by the nickase enzymes whose concentrations are set to represent the input values thereby, creating an array of solutions with DNA strands with a single nick in concentrations representing the concentrations of the nickase and therefore the values of the inputs. Next, we describe the DNA-based neural engine hardware proposed in this work followed by the execution of the basic operations for an ANN.

### 3.1 Neural engine architecture

**Fig 8:**
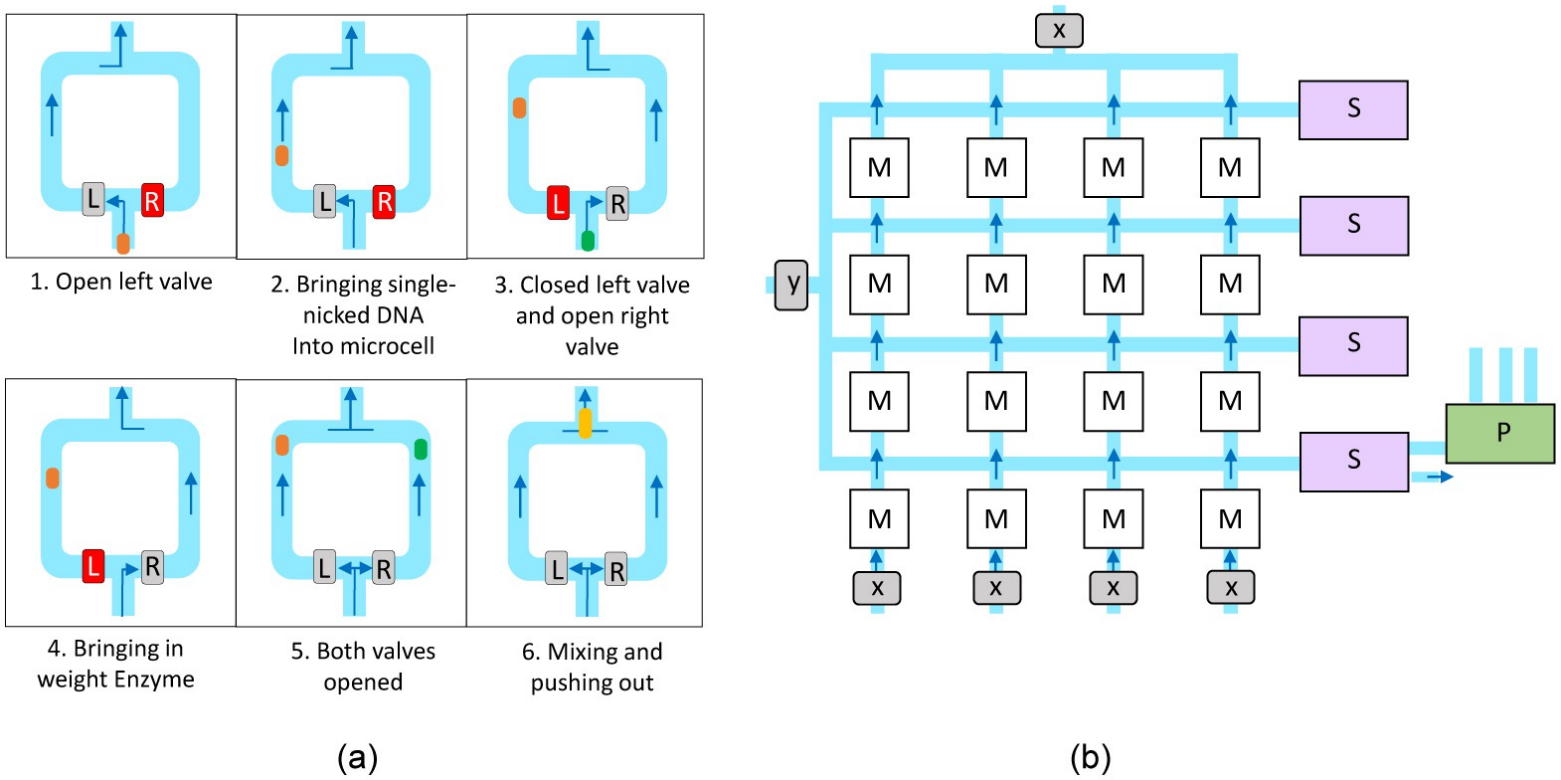
An overview of matrix multiplication execution using Microcell arrays. The Microcell operation sequence is presented in (a), with The gray and red boxes respectively represent Quake valves open and closed. The Microcell assembly for Matrix multiplication is presented in (b). The microfluidic channels are painted blue, with arrows showing flow direction induced by pressure differentiation. The M boxes are cells performing multiplications, the S boxes are responsible for droplet merging for addition operations, and the P boxes are the reaction pipelines performing activation functions. X and Y represent weights and input to the neurons respectively.

For the implementation of this process, we adopt a lab-on-chip (LoC) architecture. LoC emulates the electric signals in a digital chip with a set of controlled fluid channels, valves, and similar components. In our implementation, we will be using microfluidics where components are on the scale of 1-100 μm. Our system will operate using droplet-based microfluidics, meaning the fluid that holds data such as DNA or enzymes will move in small packages called droplets. The movement of droplets through the system will be controlled by creating pressure differentials. One critical component for controlling the flow of the microfluidic channels is the Quake valve which operates by running a pneumatic channel perpendicularly over a microfluidic channel. When the pneumatic channel is pressurized, it expands, closing the flow across the two sides of the microfluidic channel. To contain each stochastically nicked DNA droplet and merge these with weight enzymes, a small droplet storage container, which we will call a microcell, will be used as seen in Fig 8(a).

### 3.2 Microcell function

Fig 8(a) shows the sequence used to load and mix the two droplets holding the stochastically nicked DNA and weight enzymes. Throughout the loading, mixing, and release processes, there will be a constant pressure difference between the bottom and the top of the microcells shown in the figure, creating the upward flow into the next microcell. The steps, as demonstrated in Fig 8(a), are described below:

1. The right valve R is closed, and the left valve L is kept open. This has the effect of routing the fluid through the left side of the microcell, leaving the fluid on the right side static.
2. The droplet of stochastically nicked DNA enters the microcell and continues until it is known to be at a predefined, timed distance along the left channel.
3. The left valve is closed, and the right valve is opened, rerouting the fluid to flow along the right channel.
4. The weight enzyme droplet is inserted into the microcell and continuously until it is known to be approximately the same distance along the right channel. It can be observed that the DNA droplet does not move since it is in static fluid.
5. Both valves are opened, pushing both droplets simultaneously.
6. The two droplets exit the microcell together, mixing them as the channels merge.

### 3.3 Microcell assembly

The microcells will be arranged in a *k* × *k* formation, each capable of holding and mixing two droplets. These *k*^2^ microcells are interconnected with a mesh of microfluidic channels, as shown in Fig 8(b). In this figure, M, S, and P respectively represent the microcells, the merge modules, and the closing reaction pipelines. When delivering the nicked DNA droplets, all right valves are closed, and all left valves are open. The droplets are arranged at fixed distances so will travel across the microcells until each contains a single droplet. Droplet movement is controlled by introducing air flow controlled by pneumatic valves as shown in [42]. By regulating the duration of the airflow the distance of travel and separation between multiple droplets generated can be precisely controlled. The weight enzyme droplets will similarly be inserted as in steps 3 and 4 of the microcell operation, with the exception that the left and right valve states are swapped this time. All left and right valves are then opened to perform steps 5 and 6 of the microcell operation shown previously in Fig 8(a) and described in Section 3.2.

## 4 Implementation of ANN operation in the neural engine

Using the principles of stochastic computing with DNA nicking, we implement the operations involved in an ANN using the above microfluidic neural engine.

### 4.1 Execution of a multiplication in a neuron

We demonstrate the execution of a single multiplication within a microcell by mixing two droplets containing our operands. The multiplicand is a concentration of *t* DNA strands, nicked at a known site *A* at a concentration *a* (as shown in Fig 7). The multiplier *b* is represented by the concentration of nicking enzymes. The nicking enzymes are responsible for weakening the bonds holding the strands together so that after mixing and reacting, the strands nicked at both sites are our product, *a* × *b*. The multiplier is a droplet of the weight enzyme with a concentration:

$$\begin{array}{c} E=b\times t\times (1/k). \end{array}$$ (5)

Here, *k* represents the number of neurons present in the ANN layer, processed across *k* microcells and the factor 1/*k* is a consequence of distributing the nicking enzymes over k microcells. To compensate for this 1/*k* operand, each of these nicking enzymes will be given enough time to react with *k* DNA strands. This new nick will be at a second known site, *B*, near the first site *A* as shown in Fig 7. This will result in *a* × *t* of the strands nicked at site *A* and *b* × *t* of the strands nicked at site *B*. This means that the proportion of strands nicked at both sites will be the product of the two operands. A concentration of *probe strands* is then introduced to displace the small ssDNA fragment from each of the aforementioned DNA product strands, as shown in Fig 6. The resulting proportion of free-floating ssDNA fragments with respect to the total DNA (*t*) strands represents the product, *ab*.

### 4.2 Execution of dot product

The above method for scalar multiplication can be used to compute the dot product for *k* microcells, where each microcell contains the corresponding element of both input and weight vectors. Each of these *k* microcells will undergo the multiplication as described, with the multiplier, *b*, being a unique weight enzyme concentration representing the weight values for each input pair. The products in each row of the microcell array as shown in Fig 8(b) are then aggregated by mixing the droplets row-wise into one large combined droplet. This large combined droplet contains the sum of the number of fragments from each microcell which represents the dot product. Since the multiplicand in subsequent multiplications must be in the form of nicked DNA strands, this concentration of fragments must be transformed. Each fragment within the large droplet is mapped one-to-one to a nicking enzyme. This nicking enzyme is designed to nick at the primary site along a fresh, un-nicked DNA strand using a method known as strand displacement [14, 55]. The aforementioned method for dot product is implemented in the proposed microcell architecture using the following steps.

#### 4.2.1 Droplet merging

**Fig 9:**
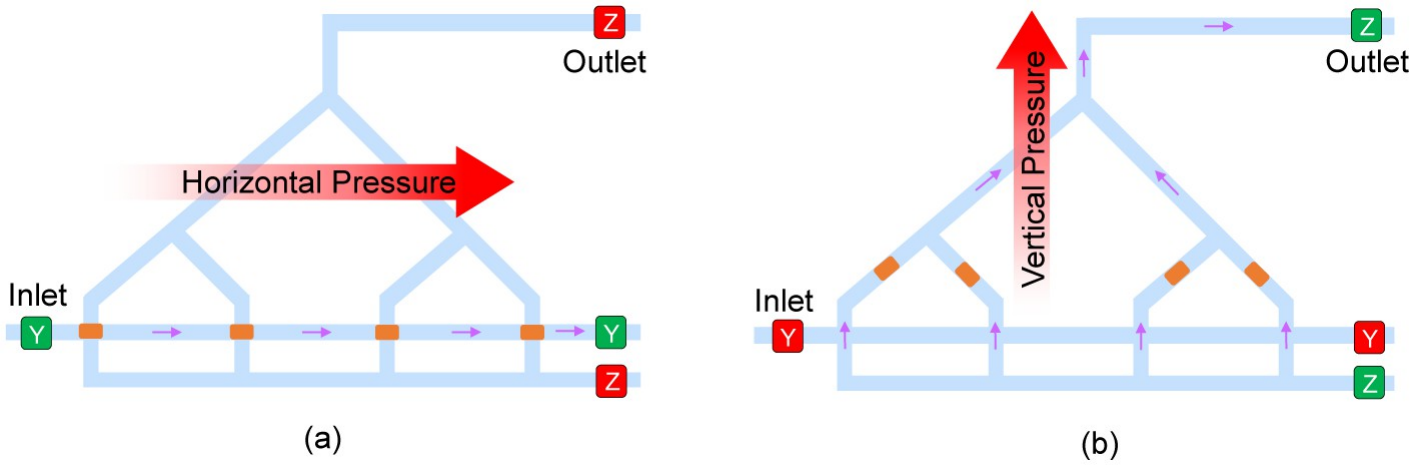
The two-step procedure for merging droplets within the Merging Module (S) for performing addition operations. The two steps include (a) rightward flow generated via closing of the Z valves and opening of the Y valves, followed by (b) upward flow generated by closing of the Y valves and opening of the Z valves.

The droplet merging module, S shown in Fig 8(b) adds the individual products of the elements of the two vectors to create the dot product. To compute the dot products as described, the mixed droplets from each microcell must be merged row-wise. Each droplet will exit the microcell, then take an immediate right turn, and remain on this horizontal path until entering the merging module, S. The two-step process is outlined as follows. Please refer to Fig 9.

1. All droplets are merged into a single large droplet with the Y valves kept open (shown in green) and the Z valves closed (shown in red). This ensures a rightward flow and no vertical pressure difference. This is shown in Fig 9(a)
2. Next, the Y valves are closed (red), and the Z valves are opened (green), causing a pressure difference that forces each droplet upward through the merge channels. The construction of the merge channels is such that each droplet reaches the final merge point at the same time. This is shown in Fig 9(b)

Once each row of droplets has been mixed, they will go through the three-step closing reaction pipeline to apply the necessary transformations as discussed below.

#### 4.2.2 Reaction pipeline

The Reaction Pipeline module enables the implementation of an activation function in the DNA Neural Engine to the previously computed dot products. In addition to implementing the activation, it also transforms the nicked fragments into a singly-nicked DNA molecule to iteratively repeat the process to implement multiple ANN layers using the following steps.

After merging all the droplets, the fraction of doubly nicked DNA molecules to all DNA molecules represents the dot product stored in the merged droplet as shown in Section 2. By applying gentle heat to this droplet, toeholds are created on DNA molecules with two nicks due to partial denaturing. The ssDNA next to this toehold can be displaced using probe strands as shown in Fig 6. Assuming complete displacement of these ssDNA molecules, the relative concentration (or to be even more precise, the relative number of molecules) of the ssDNA still represents the same fraction as the double-nicked DNA. Following this, we must apply an activation function on this ssDNA value to incorporate non-linear computations necessary in the neural networks.

Our approach utilizes a sharp sigmoid function with a user-defined transition point—i.e., the activation function is a step function with the domain and range [0, 1], and the transition point can be set in the range (0, 1). This is achieved with the DNA seesaw gates presented by Qian and Winfree [4]. This approach involves utilizing a basic DNA gate motif, which relies on a reversible strand-displacement reaction utilizing the concept of toehold exchange. The seesawing process allows for the exchange of DNA signals, with a pair of seesawing steps completing a catalytic cycle. The reader is referred to [4] for further details.

**Fig 10:**
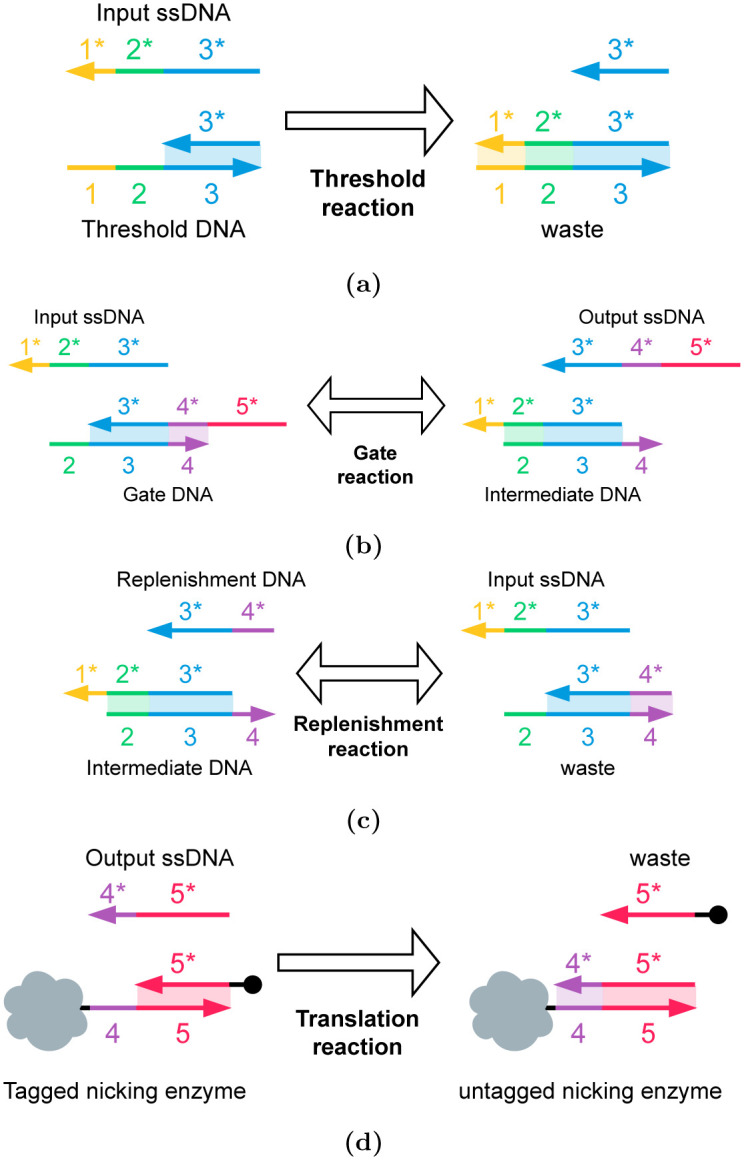
The set of reactions used to apply the activation function on ssDNA and generate an equivalent concentration of nicking enzyme. (a) The threshold reaction: The threshold molecule reacts with the input ssDNA to generate products that do not participate in any further reactions. (b) The gate reaction: the input ssDNA reacts with the seesaw gate molecule to create the output ssDNA and an intermediate molecule (c) The replenishment reaction: the replenishment strand reacts with the intermediate molecule to release more input ssDNA. This replenishes the concentration of input ssDNA and drives the production of more output ssDNA. (d) The translation reaction: the output ssDNA (domain 3* is not shown for clarity) reacts with the ‘tagged’ nicking enzyme (provided in excess) to produce an ‘untagged’ nicking enzyme. The concentration of the untagged nicking enzyme is proportional to the concentration of the output ssDNA.

We use different DNA strands for thresholding and replenishing the output. The threshold molecule binds with the input ssDNA to generate waste (Fig 10a), so the input ssDNA concentration must be larger than the threshold molecule concentration to preserve some residual amount of input ssDNA for the next stage. In the next stage, the gate reaction, the input ssDNA is used to generate output ssDNA (Fig 10b). The replenishment strand in the (Fig 10c) drives the gate reaction since it frees up more input ssDNA (Fig 10c). That is, increasing the replenishment strand concentration maximizes the concentration of the output ssDNA [4].

With these DNA reactions, a gate can be designed that applies a threshold (in detail, the input ssDNA must be greater than the threshold DNA concentration) on the input ssDNA value, and then generates an output ssDNA value of 1 due to excess replenishment molecules. This allows us to implement a sigmoid activation function. If desired, the concentration of the replenishment molecules (Fig 10c) can be limited to also apply an upper bound to the output ssDNA concentration.

1. Gentle heat is applied to the large, merged droplet. This allows the denaturing of short DNA molecules and creates toeholds.
2. A droplet containing excess probe strands is mixed to release the input ssDNA fragments. The input ssDNA is separated from the remaining molecules through the application of a magnetic field.
3. A droplet containing the DNA seesaw gate, the threshold DNA (this amount is controlled by the user-defined sigmoid function), and the replenishment DNA (in excess) molecules are mixed with the ssDNA fragments. This applies a sigmoidal activation function on the ssDNA concentration.
4. The ssDNA strands are now mapped to a specific nicking enzyme concentration. For this, a drop containing an excess of the DNA-tagged nicking enzyme will be mixed with the ssDNA. After completion of the reaction, the drop will be subjected to a magnetic field to pull down the surplus nicking enzyme molecules. The resulting solution will contain the nicking enzyme with a concentration proportional to the particular concentration of the ssDNA strands after the activation function.
5. The droplet containing the nicking enzyme is now mixed with un-nicked DNA strands to prepare the inputs to the next layer of neurons in the ANN.

After each stage of the reaction pipeline is completed, the merged droplets from each row now must be broken down into a collection of *k* smaller droplets to be entered column-wise into the microcell array. This is accomplished using a droplet separator which functions by applying a pinching pressure at some regular interval to the channels carrying merged droplets [56]. This results in a series of equally spaced droplets, which can then be placed back into the microcells column-wise.

### 4.3 Layer-wise execution of an ANN

Using the *k* × *k* array of microcells and the S and P modules an entire layer of an ANN with *k* neurons can be implemented. In this array, each column implements a single neuron of the layer, and all the columns collectively form a single layer of the ANN. All microcells in the same column contain an equal nicked DNA concentration of double-stranded DNA molecules, *A*_1_ − *A*_*k*_. The large droplet resulting from the output of each row’s activation function is now divided back into *k* originally sized droplets, which are then entered back into the microcell array column-wise, to repeat the computations for the next layer of the ANN, with the new inputs to each neuron held within the microcells.

## 5 Results

In this work, we evaluate the proposed DNA Neural Engine while processing a simple ANN using the microfluidics-based DNA computing architecture in terms of latency of processing and area footprint of the device.

The time for execution of a single layer, *t*_layer_ can be modelled as follows:

$$\begin{array}{c} t_{\text{layer}}=t_{\text{transport}}+t_{\text{mult}}+t_{\text{merge}}+t_{\text{activation}}. \end{array}$$ (6)

And,

$$\begin{array}{c} t_{\text{activation}}=t_{\text{displacement}}+t_{\text{threshold}}+t_{\text{gate}}+t_{\text{translation}}+t_{\text{nick}}. \end{array}$$ (7)

Here:

1. *t*_transport_ is the time it takes for all droplets to travel throughout the microfluidic channels for all stages in the process. It is assumed that the time taken just for transportation is not the dominant bottleneck, and so it has been estimated to be around 2 minutes.
2. *t*_mult_ is the time taken to perform a multiplication. This is the time taken for the second nicking of the strands, the second factor in the multiplication.
3. *t*_merge_ is the time taken to merge each of the small droplets per row into a single large droplet, the major step of the dot product summation.
4. *t*_activation_ can be broken up into several parts: displacement, inhibit, and nicking.
5. *t*_displacement_ is the time it takes to displace each of the ssDNA fragments from the doubly nicked strands
6. *t*_threshold_ is the time it takes for some input ssDNA strands to react with the threshold DNA.
7. *t*_gate_ is the time it takes for the displacement of the output ssDNA alongside the replenishment reaction being used to drive the gate reaction.
8. *t*_translation_ is the time it takes for ‘untagging’ the right concentration of nicking enzyme and separating it.
9. *t*_nick_ is the time it takes for the untagged nicking enzyme to react with the fresh DNA strands for the resultant node value.

The size of the proposed microfluidic device will scale quadratically with the number of neurons in a layer of the ANN, *k* to support parallel execution of all neurons. This is because any layer with *k* neurons requires an array of *k* × *k* microcells. As a pessimistic estimate, we assume each microcell will occupy an area equivalent to 6 channel widths of space in both length and breadth, given their structure with 2 microfluidic channel tracks in both horizontal and vertical directions as well an empty track for separation between the channels. Each track is assumed to be twice in width compared to the width of a channel to allow for the manufacturability of the system.

The following expression shows the area of a microcell array, *W* with *k* × *k* microcells, where *c* represents microfluidic channel width

$$\begin{array}{c} W=s\left(c\right)={\left(6kc\right)}^{2}. \end{array}$$ (8)

A pessimistic channel width of 200μm yields a resulting expression for area of (6 × 0.2 × *k*)^2^ = 1.44*k*^2^mm^2^ for the array [57]. For an optimistic estimate, assuming a channel width of 35μm, and a condensed microchamber estimate of 3 × 3 channel widths per cell, we get an area estimate of 0.01*k*^2^mm^2^ for the microcell array [57]. So depending on the manufacturing technology and fabrication node adopted, the parallelism of the device can be scaled up significantly to accommodate large hidden layers.

Table 2 shows the size and timing parameters of the microfluidic architecture [57]. Here we assume that all neurons of a single layer of the ANN can be accommodated in the device simultaneously. Using these parameters we estimate the area requirements and delay for implementation of a simple ANN capable of classifying MNIST digits. In Table 3 we show the area and delay of the ANN for various device dimensions. The area estimate considers both a pessimistic and an optimistic dimension of the microfluidic channels and chambers from a fabrication perspective. We have considered multiple configurations (Config-1 to Config-4) corresponding to different device dimensions capable of accommodating varying numbers of microcells. These configurations offer a trade-off between device size and delay in ANN processing. In Config-1, we consider the number of microcells in the microfluidic system as 196 × 196 which is capable of accommodating an ANN layer with 196 neurons. Therefore, to accommodate the input layer for the ANN that receives the 28 × 28 MNIST frames the computations are serialized by a factor of 4 to compute the whole frame. Similarly, the other configurations require serialization by factors of 16, 49, and 196, respectively. Besides the input later, the designed ANN has a single hidden layer of 784 neurons and an output layer with 10 neurons. The hidden layer is serialized with the same factor as the input layer while the output layer does not need any serialization as it has only 10 neurons except for Config-4 where it was serialized by a factor of 3. Based on the required serialization factor and due to the limited number of microcells in a die the delay of executing a single layer is modified as follows,

$$t_{layer}=((k_{layer}/k_{physical})*(t_{transport}+t_{mult}))+t_{merge}+t_{activation},$$

where *k*_*layer*_ and *k*_*physical*_ are the number of neurons in an ANN layer and the number of neurons that can be computed simultaneously on the microfluidic die respectively. The Python model of the ANN was constrained to consider only positive inputs and weights and yielded an accuracy of 96% in all the configurations as the computation model was not altered in any of them.

**Table 2:** Parameters of the microfluidic architecture.

Attribute	Value
Delay of single ANN layer (t_*layer*_)	8.07 hrs
Channel Width (Optimistic)	35μm
Channel Width (Pessimistic)	200μm
Microcell Area (Optimistic) (*W*_*min*_)	0.01*mm*^2^
Microcell Area (Pessimistic) (*W*_*max*_)	1.44*mm*^2^

**Table 3:** Summary of the estimated system performance.

Configuration	# Microcells/Die	Microcell Array Area Pessimistic (cm^2^)	Microcell Array Area Optimistic (cm^2^)	Execution Time/Layer (hrs.)
Config-1	196 × 196	553.19	3.84	14.17
Config-2	49 × 49	34.57	0.24	38.6
Config-3	16 × 16	3.69	0.03	105.6
Config-4	4 × 4	0.23	0.002	404.6

We use a sigmoid activation function in all the layers, implemented with ‘seesaw’ gates [4], as discussed above. This enables signal amplification in the form of a sigmoid function—precisely what we need. Again, the reader is referred to [4] for further details.

We assume that the partial results of the serialized computation can be stored in the DNA solution medium in an external reservoir array [58] that is communicating with the microfluidic ANN system through a microfluidic bus interface where the reservoirs are indexed and routed using the valve-system of the microfluidic system to the appropriate micro-chamber corresponding to the appropriate neuron.

Note that a configuration that minimizes the computational delay of the ANN for MNIST classification evaluated here would need a system with an array of 784 × 784 microcells to accommodate the entire input layer simultaneously. However, that would make the die size unrealistic. Therefore, such a system could consist of multiple smaller microfluidic dies integrated on a microfluidic interposer substrate capable of communicating between the dies enabling a scalable solution [59]. This system with 784 × 784 microcells would reduce the delay per layer of the ANN to 8.07 hours.

After evaluating the proposed DNA-based LoC Neural computing engine, we have also extended the result for a more complex task. Since a very notable targeted application of the proposed work is archival data search, matching, or retrieval, both digit and text recognition are significant tasks. While the digit recognition application has been already covered with our evaluation of the MNIST classification task, as a further extension, we also are evaluating the proposed system for text recognition. In this regard, we have chosen a four-layer MLP architecture presented in [60] for text recognition applications. Based on our analytical models for delay and area, this application will require 12.46 hours of execution time for this MLP, with 4*cm*^2^ of LoC area while achieving an accuracy of 90%.

A distinct advantage of using the DNA-based approach is that the variability of DNA as a computing medium adds an interesting new factor to ANN training. Slight variations in any reaction in the process could be used as a natural source of drift in training. Iterative feedback from executing the model could be used to correct the errors and further train the model indefinitely. This is not something reflected in traditional digital implementations without the artificial introduction of variation or noise between the models.

### 5.1 Efficiency of DNA TMSD reactions

High efficiency of toehold mediated strand displacement reactions is vital for developing our microfluidics device. Several factors influence this efficiency. Here we address these numerous points:

1. Molecule concentration: We use the relative amount of nicked DNA to store variables. For values close to 0, we operate with a small concentration of nicked DNA molecules. These molecules will yield few ssDNA strands for downstream reactions. This can result in larger relative errors and quantization errors. One solution to this is to use high concentrations of DNA molecules before nicking.
2. Reaction rate: The rate of reactions is dependent on the concentrations of all reactants. Once again, small values are stored in lower concentrations of DNA molecules. Computing on these values would require longer reaction times. The output DNA has to be sampled after the reaction product concentration has converged for all possible input values.
3. Reaction conditions: Conditions such as pH and temperature not only influence DNA stability but also reaction kinetics and enzyme efficiency. In our system, we use an increase in temperature to drive DNA denaturing. Increasing the temperature can be used to speed up displacement reactions, but has to be done with measures to minimize DNA degradation. We also manipulate nickase concentration to drive nicking in target DNA molecules in our scheme. These enzymes must be carefully separated after their nicking is done to ensure they do not interfere with DNA strand displacement reactions.
4. Competing interactions: Having various different DNA molecules in a solution can lead to unintended reactions. For example, a DNA molecule can trigger a strand displacement reaction on a molecule besides its intended target. This is called *leakage*. It can result in input concentration of ssDNA being ‘lost’ to an unintended target, or an erroneous ‘gain’ in output DNA concentration from the wrong input. Other issues such as the formation of secondary structures can also interfere with strand displacement. To minimize these issues, DNA molecules must be synthesized to be orthogonal to each other, and solutions should not contain unnecessary DNA molecules that are not involved in the present reaction [61].
5. Implementation challenges: Operating on a microfluidics scale presents its own challenges to DNA strand displacement reactions. Consider the separation of DNA molecules using magnetic beads. The magnetically beaded molecules can be held in place with a magnetic field, while the remaining molecules can be washed off. This process must be done carefully—gently enough to prevent stress that could break the immobilized DNA molecules, yet with enough force to ensure complete separation. Even in the case of digital microfluidics, factors such as evaporation can change DNA concentration in small droplets and impact up reaction results.

## 6 Conclusions

Conventional silicon computing systems generally have centralized control with a CPU that can aggregate sensory data, execute arbitrarily complex analysis, and then actuate. For molecular applications, the actions of sensing, processing, and actuating must all be performed *in situ*, in a decentralized way. Our goal in this paper was to devise molecular computing in which data processing occurs in the storage system itself using the natural properties of the molecules, with no need for readout and external electronic processing. *In situ* molecular processing of data is critical from the standpoint of I/O: reading and writing data will always be a bottleneck for molecular systems. Computing “in-memory” is, therefore, a prerequisite.

DNA emerges as a dense substrate for data storage, while neural networks prove to be valuable tools in bioinformatics [62, 63]. Consequently, various studies have explored the design of neural network architectures within the context of DNA computing [64, 65]. These approaches often rely on cascades of toehold-mediated DNA strand displacement reactions to model arithmetic operations. Our approach shares similarities in this regard, but it comes with distinct advantages. First, our data is stored stochastically, enabling computation across a continuous range of values from 0 to 1, as opposed to being restricted to binary values as typically seen in digital logic. This reduction in complexity benefits operations like multiplication. Second, our computation takes place within a single lab-on-a-chip device. The utilization of microfluidics enhances parallelization and automation of reactions, while also providing a portable platform.

Another means of achieving such computation would be through the use of digital microfluidics [66, 67]. These devices boast faster droplet processing and smaller operating volumes over continuous flow microfluidics devices. Indeed, we are collaborating with an industrial partner, Seagate, on the development of digital microfluidics technology for DNA storage [68]. However, this technology presents its own limitations. These include the difficulty of breaking apart or merging droplets precisely, and the need for high voltages which could hinder future biocompatible applications. Digital microfluidics will take many years to mature. However, when does, techniques for computing *on* the data that is stored in DNA will be needed. While conceptual in nature, this paper demonstrates how such computation could be performed.

This paper presented a methodology for implementing complex operations, including ANN computation, on data stored in DNA. The paper weaves together two distinct strands: a conceptual representation of data, on the one hand, and the technology to compute with this representation, on the other hand. The representation is a fractional encoding on the concentration of nicked DNA strands. With this representation, we can compute a *fraction* of a *fraction*—so the operation of multiplication—borrowing ideas from stochastic logic. The “read-out” process is effected by releasing single strands via DNA toehold-mediated strand displacement. The technology is microfluidics. We described the microcell layout used in a pneumatic lab-on-a-chip (LOC) to control mixing of DNA solutions. Mixing allows us to compute a fraction of a fraction of a concentration value. Based on this core operation, we presented a full architecture to implement neural computation.

There are a number of practical challenges. One of the concerns ubiquitous with DNA strand displacement operations is “leakage”, that is to say errors in transforming concentrations. Based upon the actual experimental results, we might have to mitigate leakage with error correction methods or adopt so-called “leakless” designs [69].

In future work, we will investigate ambitious applications of small molecule storage and computing. Our goal is to devise *in situ* computing capabilities, where sensing, computing, and actuating occur at the molecular level, with no interfacing at all with external electronics. The applications include:

- **Image processing and classification**: We will implement a full-scale molecular image classifier using neural network algorithms. Performing the requisite image processing *in situ*, in molecular form, eliminates data transfer bottlenecks. We will quantify the accuracy of image processing in terms of the *signal-to-noise* ratio and the *structural similarity index*.
- **Machine learning**: We will explore a common data representation for integrating sensing, computing, and actuation *in situ*: hyperdimensional random vectors. Data is represented by long random vectors of integer or Boolean values. We will deploy this paradigm for machine learning, exploiting the randomness of molecular mixtures for encoding, which can naturally map to large vector representations.
